# Feasibility of ultrasound-guided sacroiliac joint puncture in the treatment of inflammatory and osteoarthritis pain: a 11-years single-centre experience

**DOI:** 10.3389/fmed.2026.1997507

**Published:** 2026-10-22

**Authors:** Carlos A. Guillén-Astete, Samantha Kanaffo, África Andreu-Suarez, Claudia Urrego-Laurín, Boris Blanco-Cáceres, Pablo A. Zurita-Prada

**Affiliations:** 1 Rheumatology Department, Ramón y Cajal University Hospital, Madrid, Spain; 2 Servicio de Reumatología, Hospital Universitario Asisa Moncloa, Madrid, Spain; 3 Department of Medicine, Faculty of Medicine, Health and Sports, Universidad Europea de Madrid, Madrid, Spain; 4 Rheumatology Department, Blua Valdebebas Sanitas Hospital, Madrid, Spain

**Keywords:** corticosteroid injection, joint puncture, osteoarthritis, sacroiliac joint (SIJ), spondyloarthritis, ultrasound

## Abstract

**Background:**

Sacroiliac joint inflammation is a key manifestation of spondyloarthritis; however, most sacroiliac pain arises from mechanical overload, which also involves an inflammatory component. Ultrasound-guided sacroiliac joint puncture (SJP) for local corticosteroid injection is a semi-invasive procedure infrequently performed in rheumatology clinics, despite previous reports describing its feasibility and relative simplicity.

**Objective:**

To describe the clinical outcomes and safety of ultrasound-guided sacroiliac joint puncture (SJP) in patients with osteoarthritis (OA) or spondyloarthritis (SpA), and to identify independent predictors of clinical improvement.

**Methods:**

We conducted a retrospective study including 130 patients who underwent 155 ultrasound-guided SJP procedures. Demographic variables were analysed per patient, whereas clinical outcomes were analysed per procedure. Improvement, no change/no report and worsening were obtained from follow-up clinical records. A complementary patient-level analysis restricted to the first procedure per patient was performed. Multivariable logistic regression was used to evaluate independent predictors of improvement.

**Results:**

The mean age of the cohort was 48.7 years. Clinical improvement was observed in 130 procedures (83.9%), 23 (14.8%) showed no change or no report, and 2 (1.3%) were associated with worsening. Improvement rates differed significantly between diagnostic groups: 75.6% in OA and 92.2% in SpA (*p* < 0.01). In the multivariable model, diagnosis was the only independent predictor of improvement, with SpA showing higher odds of response compared with OA (adjusted OR 4.22, 95% CI 1.22–14.58, *p* = 0.023). Obesity, fibromyalgia, age and sex were not independently associated with outcome. The patient-level analysis yielded similar results.

**Conclusion:**

Ultrasound-guided SJP is a safe procedure associated with high rates of clinical improvement. Patients with SpA show significantly greater benefit than those with OA, and this association remains robust after multivariable adjustment. Underlying inflammatory disease appears to be a key determinant of therapeutic response.

## Introduction

Sacroiliac joint puncture (SJP) is a procedure performed to administer corticosteroids or other therapeutic agents directly into the sacroiliac joint, or to obtain synovial fluid for diagnostic purposes (1). The procedure is considered a useful intervention for managing sacroiliac pain associated with mechanical overload or osteoarthritis, as well as for controlling local inflammatory manifestations in patients with spondyloarthritis (2–4).

Owing to the deep location and oblique orientation of the sacroiliac joint, blind puncture based solely on anatomical landmarks is technically challenging and often unreliable. This difficulty stems from its oblique orientation relative to the skin plane and its proximity to the sacral irregular surfaces (5, 6). Even the most accessible cephalic portion of the joint may lie several centimetres beneath subcutaneous tissue, particularly in patients with a high body mass index. This additional obstacle limits the use of a standard 30 mm needle and may require the procedure to be performed with longer devices, such as a lumbar puncture needle.

Image guidance substantially improves the accuracy and safety of SJP. Techniques such as computed tomography, magnetic resonance imaging and fluoroscopy have been used to assist needle placement (4, 7–9); however, these modalities may not be readily available in routine clinical practice. Ultrasound offers an accessible, radiation-free and immediate alternative that enables clinicians to perform SJP without delays associated with scheduling radiological procedures.

Our centre has routinely performed ultrasound-guided SJP since 2012, with two rheumatologists trained in musculoskeletal ultrasound and interventional techniques carrying out the procedures. The aim of this study is to describe our 10-year single-centre experience, summarising indications, technical aspects, clinical outcomes and safety observations.

## Materials and method

Our centre has performed ultrasound-guided sacroiliac joint puncture (SJP) since 2012. For the present study, we conducted a retrospective review of all patients who underwent this procedure between June 2015 and June 2025, a period during which electronic clinical records provided systematic access to clinical information. Two researchers independently reviewed the records following a standardised data-collection protocol.

### Patient data and clinical variables

Demographic and clinical data were extracted from electronic records, including underlying diagnosis, indication for the procedure, technical details, clinical outcomes and adverse events. Obesity was defined as BMI ≥30 kg/m^2^. BMI was calculated when height and weight were available, and patients meeting this threshold were classified as obese. Fibromyalgia was diagnosed according to American College of Rheumatology (ACR) 2010/2016 criteria, based on clinical documentation in the medical record.

Demographic variables (age, sex, diagnosis, obesity and fibromyalgia) were analysed per patient (*n* = 130), whereas clinical outcomes (improvement, no change/no report, worsening, repeated procedures) were analysed per procedure (*n* = 155).

All patients underwent MRI of the sacroiliac joints, which served as the primary diagnostic tool to attribute pain to the joint and to discriminate between osteoarthritis and spondyloarthritis. MRI findings were reviewed by experienced musculoskeletal radiologists. Degenerative sacroiliac joint disease was defined by joint space narrowing, subchondral sclerosis, osteophytes and irregularity of the articular margins. Active sacroiliitis was defined according to ASAS criteria (bone marrow oedema on STIR sequences in typical locations).

All patients with spondyloarthritis fulfilled ASAS classification criteria for axial or peripheral disease.

### Procedure details

All procedures were performed by two rheumatologists with expertise in musculoskeletal ultrasound and ultrasound-guided interventions. Verbal informed consent was obtained prior to each procedure, in accordance with institutional protocols for minor ultrasound-guided techniques.

Needle selection varied according to patient habitus and the estimated skin-to-joint distance. Standard procedures were performed using 21-gauge needles of 30–40 mm, whereas 20-gauge lumbar puncture needles of 90 mm were used in patients with a high body mass index or when the skin-to-joint distance exceeded 30–40 mm. The total injected volume did not exceed 1 mL. Triamcinolone (20 mg in 0.5 mL) or betamethasone (5.7 mg in 0.5 mL) was administered, with optional addition of a local anaesthetic in cases of severe pain. In our centre triamcinolone was used in all patients except in three cases in which triamcinolone was not available.

### Outcome assessment

Clinical outcome was assessed using two sources: the treating physician's global impression of improvement, and changes in pain intensity measured using a 0–10 visual analogue scale (VAS).

Post-procedure assessment was performed at the first follow-up visit (4–12 weeks). Improvement was defined as either a positive clinical judgement by the treating physician or a reduction in VAS score. “No change” was defined as stable symptoms, and “no report” indicated absence of documented outcome.

Clinical examination included standardised sacroiliac provocation tests (FABER, Gaenslen, thigh thrust, compression/distraction). Differential diagnosis with facet joint pain, discogenic pain and radicular pain was considered in all cases.

### Follow-up and adverse events

The observation period for each procedure extended to 12 months after the intervention. Adverse events were considered procedure-related if reported within 90 days following SJP, regardless of the date of documentation in the clinical record.

## Results

### Demographic and clinical data

A total of 130 patients underwent 155 ultrasound-guided sacroiliac joint puncture (SJP) during the study period: 78 were performed over patients diagnosed by sacroiliac osteoarthritis and 77 over patients with spondyloarthritis. In two patients, MRI demonstrated coexistence of degenerative and inflammatory changes. Based on clinical correlation and fulfilment of ASAS criteria, both were classified within the spondyloarthritis group. Of the 155 procedures, 30 were repeated interventions performed in 25 patients. Five procedures were bilateral and were carried out during the same session. Demographic variables were analysed per patient, as shown in Table 1. Mean age (standard deviation) of patients at the time of procedures was 48.7 (6.3), with a range of 29 to 81. The most prevalent diagnosis related to the indication of SJP was osteoarthritis, and was present in 78 procedures (50.3%), obesity and fibromyalgia also were present and distributed asymmetrically among patients with and without an inflammatory underlying joint disease (Table 1).

**Table 1:** Demographic and clinical data of the cohort of procedures analysed.

Diagnosis	Sex		Mean age	BT	JAK-i
	Male	Female			
OA	7 (36.8%)	8 (13.6%)	65.6		
OA + obesity	10 (52.6%)	11 (18.6%)	49.4		
OA + obesity + fibromyalgia	1 (5.3%)	24 (40.7%)	48.6		
OA + fibromyalgia	1 (5.3%)	16 (27.1%)	51.0		
Total OA	19 (24.3%)	59 (75.6%)	52.6 (7.6)		
SpA	28 (36.4%)	8 (24.2%)	45.5	13	
SpA + obesity	15 (19.5%)	9 (27.3%)	44.6	12	1
SpA + obesity + fibromyalgia	1 (1.3%)	14 (42.4%)	42.8	8	1
SpA + fibromyalgia	0 (0.0%)	2 (6.1%)	42	2	
Total SpA	44 (57.1%)	33 (42.8%)	44.6 (6.4)	35	2

Obesity was considered when BMI was available, or when overweight or obesity was explicitly noted in the physical examination in the clinical records.

SpA, Spondyloarthritis-related diagnostics, including axial-SpA, psoriatic-SpA, IBD-related SpA and reactive arthritis.

BT, Patients treated with a biological therapy; JAK-i, Patients treated with a JAK inhibitor; OA, osteoarthritis or overload reported clinically and supported by radiological means.

Of the 77 cases of spondyloarthritis (49.7%), 63 had axial spondyloarthritis (81.8%), 9 had psoriatic arthritis (11.7%), 3 had inflammatory bowel disease-related entesopathy, and 2 had reactive arthritis. At the time of the SJP, 35 patients were receiving biological therapy (28 with an anti-TNF-α agent) and 2 were receiving a JAK inhibitor. Four patients were under treatment with acenocoumarin and three with dabigatran. No patient had been receiving heparin.

### Procedure

To perform SJP, the patient must lie face down on a rigid stretcher. The technique for performing the procedure, also known as the upper approach (2, 3, 10), was used in all cases, with slight modifications. The skin from the second lumbar spine to the middle of the buttocks must be exposed. Using a linear probe set to a frequency between 6 and 9 MHz, the spinous process of the fifth lumbar vertebral body is identified. Using this cortex as a reference, the probe is displaced 1 to 2 cm in the caudal direction and 2 to 3 cm to the right or left, depending on the target sacroiliac joint. At this point, the probe should be slightly rotated to compensate for the disposition of the joint's upper and posterior aspects. Once the desired sacroiliac joint is identified and the skin surface is properly sterilised, the operator will introduce the needle along the long axis of the probe, from medial to lateral, with an inclination angle of 30 to 45 degrees until the bezel reaches the joint (Figure 1).

**Figure 1:**
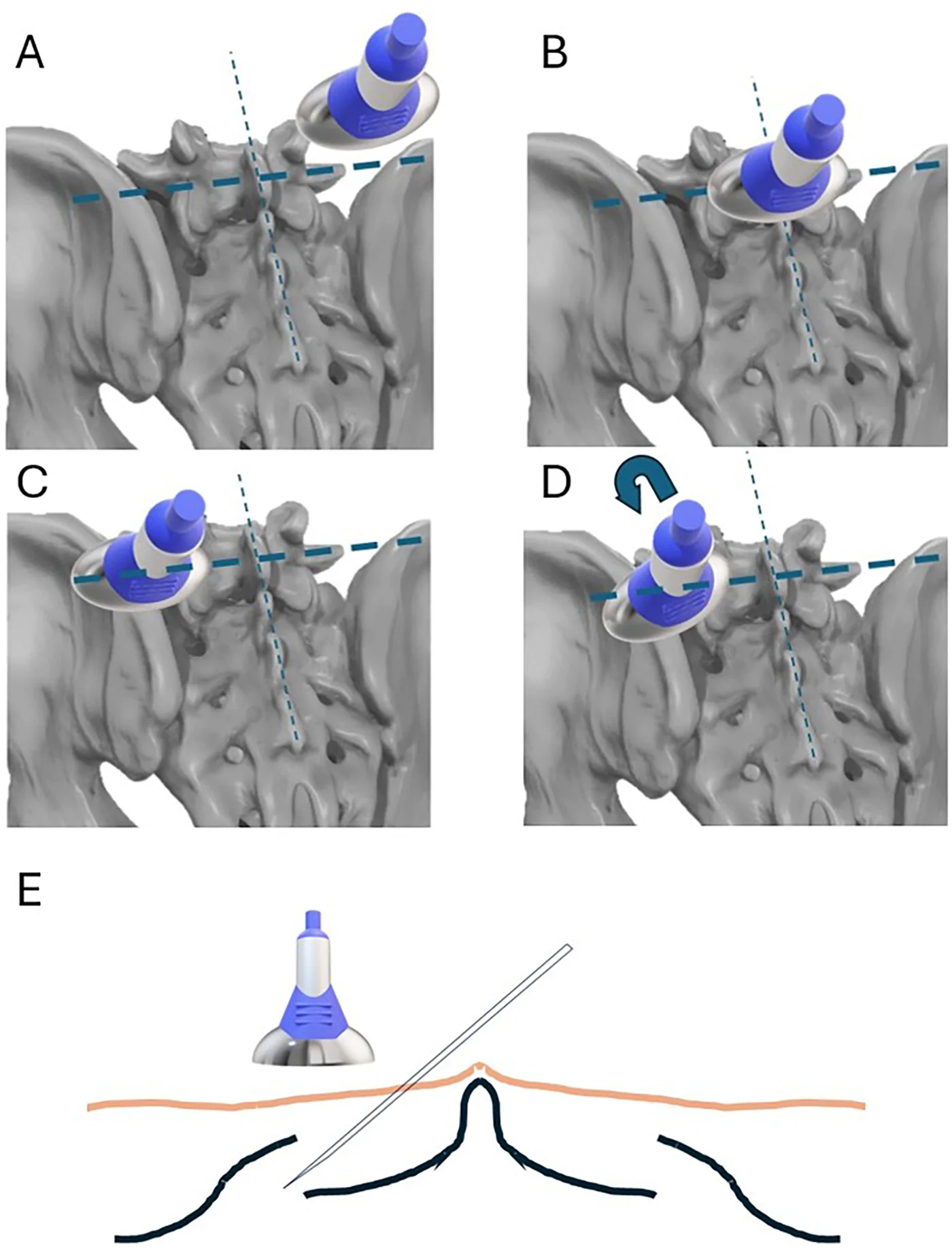
Schematic overview of the ultrasound-guided sacroiliac joint puncture. **(A,B)** Probe placement over the fifth lumbar spine in a transverse approach over the spine axis; **(C)** Lateral displacement of the probe until the cortical surface of the iliac crest is reached; **(D)** Slight rotation of the probe to align its longitudinal axis perpendicular to the joint; **(E)** Disposition of the needle over the joint using the plane of the probe with a angle of access of 30 to 45 degrees, from medial to lateral.

Based on the distance from the skin to the joint, the needle length will be chosen. An intramuscular needle (30 mm) can be used in people with a low body mass index or a distance of 2 cm. With superior distances, a lumbar puncture needle should be considered.

The total volume to be injected must not exceed 1 cc. The corticosteroid used can be triamcinolone (20 mg in 0.5 mL) or betamethasone (5.7 mg in 0.5 mL). The use of an anaesthetic mixed with the corticoid fluid is acceptable in cases of severe or acute pain, considering that the sacroiliac joint capsule is not very permeable due to the fibrous nature of its capsule. In our cohort, no patient underwent an SJP to inject any substance other than corticosteroids with or without anaesthetics. In no case were procedures indicated for collecting a joint fluid sample or for other diagnostic purposes.

The procedure was performed in 10 to 15 min. In no case did the procedure have to be suspended or delayed due to pain during the puncture or any other cause; however, in 6 cases (3.9%), the patient experienced pain severe enough to require up to 5 min of observation after the procedure.

Anticoagulant therapy was managed according to institutional safety protocols for low-risk musculoskeletal interventions.

Patients receiving acenocoumarol underwent INR testing prior to the procedure. Injections were performed only when the INR was ≤3.0, and anticoagulation was not interrupted.

Patients treated with dabigatran were assessed according to renal function. Dabigatran was withheld for 24 h before the procedure and restarted the following day.

### Outcomes

One hundred and thirty procedures were considered successful by the corresponding faculty (83.9%) during the follow-up period Table 2. Thirty patients underwent a second SJP in the same joint (15 procedures) or in the contralateral joint. In 23 procedures (14.8%), the outcome was not reported (16 procedures), or the pain level did not change (7 procedures); in 2 cases (1.3%), a worsening was reported. The mean interval between two procedures in the same patient was 110 (11) days. In 58 procedures, the VAS pain scores before and after the SJP were recorded, and a mean reduction of 5.7 (3.0) points was observed. Improvement rates differed significantly between diagnostic groups at the procedure level: 75.6% in osteoarthritis and 92.2% in spondyloarthritis (*χ*^2^ test, *p* < 0.01). Similar differences were observed in the patient-level analysis restricted to the first procedure per patient, with spondyloarthritis consistently showing higher success rates.

**Table 2:** Summary of outcomes in the cohort.

Diagnosis	Clinical outcome			VAS mean change	Repeated procedure
	Improv.	No change/no report	Worsening		
OA	15 (100%)	–	–	−6.4	1
OA + obesity	12 (57.1%)	9 (42.9%)	–	−6.3	1
OA + obesity + fibromyalgia	18 (72%)	6 (24%)	1 (4%)	−3.2	1
OA + fibromyalgia	14 (82.4%)	2 (11.8%)	1 (5.9%)	NDA	–
Total OA	59 (75.6%)	17 (21.8%)	2 (2.6%)	−4.9	3
SpA	33 (91.7%)	3 (8.3%)	–	−6.8	12
SpA + obesity	21 (87.5%)	3 (12.5%)	–	−4.9	10
SpA + obesity + fibromyalgia	15 (100%)	–	–	−4.2	5
SpA + fibromyalgia	2 (100%)	–	–	NDA	–
Total SpA	71 (92.2%)	6 (7.8%)	–	−6.0	27

Overall clinical outcome was obtained directly from the clinical annotations in the follow-up visit after the procedure. Percentages have been calculated from the original number of patients who underwent the SJP, based on the underlying diagnosis. Outcome was based on physician assessment and/or VAS change. VAS change was calculated with available data (58 cases). NDA: No data available. OA: osteoarthritis or overload reported clinically and supported by radiological means.

VAS measured on a 0–10 scale; post procedure assessment at 4–12 weeks.

SpA, Spondyloarthritis-related diagnostics, including axial-SpA, psoriatic-SpA, IBD-related SpA and reactive arthritis; VAS, Visual analogue scale of pain.

In the multivariable logistic regression model, diagnosis remained the only independent predictor of clinical improvement. Patients with spondyloarthritis had significantly higher odds of improvement compared with those with osteoarthritis (adjusted OR 4.22, 95% CI 1.22–14.58, *p* = 0.023).

Obesity showed a non-significant trend towards lower improvement (OR 0.36, 95% CI 0.12–1.13, *p* = 0.081), whereas fibromyalgia showed a non-significant trend towards greater variability in outcomes (OR 2.08, 95% CI 0.69–6.27, *p* = 0.192). Age and sex were not associated with clinical response.

The patient-level analysis restricted to the first procedure per patient yielded similar results, confirming the robustness of the association between diagnosis and clinical improvement.

### Safety

No severe adverse event was reported in our cohort; however, one patient had to be admitted to the hospital on suspicion of infection; this diagnosis was ruled out following two sterile joint aspirations. No bleeding events or haematomas were observed in any patient included those who were anticoagulated.

No specific prophylactic analgesic recommendation was made after procedures; however, in 10 cases, short-term anti-inflammatory treatment (less than a week) was noted. Pain reported immediately after the procedure in 6 cases was not associated with short- or long-term outcomes and was considered part of the expected course, as previously reported. All patients declared that they would undergo the procedure again if necessary.

## Discusion

In our experience, SJP is a well-tolerated procedure with a high rate of clinical success in improving pain in cases of sacroiliac pain due to osteoarthritis or spondyloarthritis. In patients with spondyloarthritis and sacroiliac pain as a unique uncontrolled symptom, we can hypothesize that SJP could delay the need to change to an advanced therapy (biological or JAK-inhibitors). The clinical improvement observed in our cohort aligns with previous studies reporting favourable outcomes after intra-articular corticosteroid injection for sacroiliac joint pain (12). In spondyloarthritis, several authors have described meaningful short-term benefit, particularly in patients with active sacroiliitis on MRI, whereas improvement in mechanical or degenerative pain tends to be more variable (13, 14). Our results reflect this pattern, with significantly higher odds of improvement in patients with spondyloarthritis compared with those with osteoarthritis.

The accuracy of ultrasound-guided sacroiliac joint injection has been evaluated in cadaveric and imaging-correlated studies, which show that ultrasound improves needle placement relative to blind techniques but cannot confirm intra-articular spread with the same certainty as fluoroscopy or CT (9, 11, 15). This limitation applies to our study and has been acknowledged. Nevertheless, the high rate of clinical improvement suggests that periarticular or intraligamentous deposition may still provide therapeutic benefit, as previously reported.

The multivariable analysis demonstrated that underlying diagnosis was the only independent determinant of clinical improvement. Patients with spondyloarthritis were more than four times as likely to improve as those with osteoarthritis, even after adjusting for obesity, fibromyalgia, age and sex. This reinforces the notion that inflammatory sacroiliac pain responds more consistently to ultrasound-guided sacroiliac joint puncture than mechanical or degenerative pain.

Obesity and fibromyalgia did not emerge as independent predictors of outcome, although fibromyalgia was associated with greater subjective variability. Age and sex had no measurable effect. The consistency between procedure-level and patient-level analyses supports the validity of these findings despite the presence of repeated and bilateral procedures.

The diagnostic process was strengthened by the fact that all patients underwent MRI, allowing reliable discrimination between degenerative and inflammatory sacroiliac joint disease. MRI also identified two cases with mixed degenerative and inflammatory features; both fulfilled ASAS criteria and were classified as spondyloarthritis.

Although BASDAI and ASDAS scores were not collected, classification according to ASAS criteria and MRI findings provided robust diagnostic certainty. Differential diagnosis with facet joint pain, discogenic pain and radicular pain was systematically considered, and lumbar imaging was reviewed when appropriate.

The cephalic approach used in our centre is based on anatomical and safety considerations. Although the posterosuperior portion of the sacroiliac joint contains a higher proportion of ligamentous tissue, it is also the most superficial segment and provides a reliable acoustic window with minimal needle travel. In contrast, approaches targeting the inferior pole expose the needle trajectory to the vicinity of the sacral foramina, increasing the risk of inadvertent neurovascular contact.

Despite the ligamentous composition of the cephalic portion, needle advancement is generally straightforward, and the low resistance encountered during injection is consistent with deposition within the joint line or immediately adjacent to it. Owing to the anatomical continuity of the sacroiliac joint, injectate delivered into the cephalic region spreads along the articular space and reaches the synovial compartment.

As with all ultrasound-guided sacroiliac joint procedures, intra-articular spread cannot be confirmed directly, and we have moderated statements implying guaranteed intra-articular delivery. Nevertheless, the high rate of clinical improvement observed in our cohort supports the therapeutic relevance of this approach.

Importantly, the absence of a control group prevents definitive conclusions regarding efficacy. Placebo effect, regression to the mean, natural history of sacroiliac pain and concomitant treatments may have contributed to the observed improvement. These factors must be considered when interpreting the results.

Intra-articular corticosteroid injection forms part of a broader treatment algorithm for chronic sacroiliitis, ranging from conservative management (physiotherapy, NSAIDs) to interventional procedures such as radiofrequency denervation and, in refractory cases, surgical fusion. A recent review (16) highlight the role of image-guided injection as a minimally invasive option that may provide short-term relief and facilitate rehabilitation. Our findings support its use as part of a multimodal strategy, particularly in patients with inflammatory sacroiliitis.

The safety profile observed in our cohort is consistent with previous reports of ultrasound-guided sacroiliac joint injections, which describe a low rate of complications (12). In our series, no patient experienced any complications, although there was one suspected case of infectious arthritis which was ultimately ruled out. As regards any specific discomfort experienced during the procedure itself, our cohort of patients, in line with other series (17), showed no reluctance to undergo the procedure again should it be necessary.

We also acknowledge that retrospective retrieval of adverse events from clinical documentation may underestimate mild or systemic corticosteroid effects such as transient hyperglycaemia, facial flushing or vasovagal reactions. No major complications were identified, but the possibility of under-reporting of minor events must be considered.

The retrospective design of the present study is its most obvious weakness. It is highly probable that the overall improvement assessed by the faculty included even slight changes in pain that were not necessarily relevant to the patient. Besides, the utility of the SJP in delaying or avoiding a change in a specific therapy was not assessable given the particularities of a retrospective clinical history review-based study. Finally, the lack of an extended VAS evaluation of changes in pain prevents us from accurately determining the effect on the most relevant variable to order an SJP. In order to better understand the effect of this procedure in patients with or without underlying inflammatory disease, a longitudinal prospective study should be conducted.

## Data Availability

The raw data supporting the conclusions of this article will be made available by the authors, without undue reservation.
